# Cyclosporine A and Romiplostim as a Bridging Therapy to Curative Surgery in Aplastic Anemia Complicated by Gastric Cancer: A Case Report

**DOI:** 10.1002/jha2.70440

**Published:** 2026-10-21

**Authors:** Noriharu Nakagawa, Masahide Yamazaki, Kohei Hosokawa

**Affiliations:** ^1^ Department of Hematology Keiju Medical Center Nanao Japan; ^2^ Department of Hematology Faculty of Medicine Institute of Medical Pharmaceutical and Health Sciences Kanazawa University Kanazawa Japan

**Keywords:** aplastic anemia, bridging therapy, case report, gastric cancer, romiplostim

## Abstract

We present a case of aplastic anemia (AA) complicated with gastric cancer that was successfully treated with cyclosporine A (CsA) and romiplostim (ROMI) as a bridging therapy before curative surgery. This patient had untreated gastric cancer at the time of AA diagnosis, and surgery was difficult because of thrombocytopenia. However, CsA and ROMI were effective, resulting in a rapid increase in the platelet count, thereby making surgery for gastric cancer possible. CsA and ROMI as a bridging therapy to curative surgery may therefore represent a feasible treatment option in patients with AA complicated by malignant tumors.

Trial Registration: The authors have confirmed clinical trial registration is not needed for this submission.

## 1 Introduction

Aplastic anemia (AA) is characterized by pancytopenia and bone marrow (BM) hypoplasia [1]. Primary acquired AA is largely an autoimmune BM failure caused by a cytotoxic T‐lymphocyte attack against hematopoietic stem cells [1]. Patients with primary AA can be successfully treated with immunosuppressive therapy (IST) such as anti‐thymocyte globulin (ATG) and cyclosporine A (CsA). Recently, thrombopoietin receptor agonists (TPO‐RAs), including eltrombopag (EPAG) and romiplostim (ROMI) have shown efficacy against AA in combination with IST [2, 3]. Therefore, the treatment options for AA are increasing in clinical practice.

It is important to note whether patients with AA also have malignant tumors because there is some potential concern that IST increases the risk of cancer aggravation via the suppression of the immune surveillance of cancers. In particular, in cases of severe AA, clinicians must carefully decide whether to use ATG, given its pan T‐cell suppressive effects. The role of TPO‐RAs in AA with malignancy remains unclear.

In this report, we present a case of AA complicated with Sjögren's syndrome (SS). This patient had gastric cancer that required surgical resection at the time of the AA diagnosis. Although the patient had significant thrombocytopenia, the platelet counts rapidly improved with CsA and ROMI, thereby allowing the patient to safely undergo surgery.

## 2 Case Report

A 76‐year‐old woman presented to our hospital with the chief complaint of dry mouth. The patient was diagnosed with SS, and symptomatic treatment for dryness was thus initiated. Approximately one year after the diagnosis of SS, her anemia progressed, and endoscopy revealed gastric cancer. Thrombocytopenia progressed, resulting in pancytopenia. Her BM showed hypoplasia with decreased megakaryocytes; no significant dysplasia or an increase in blasts was observed. A chromosomal analysis of the BM revealed a normal karyotype. Peripheral blood revealed 0.007% paroxysmal nocturnal hemoglobinuria (PNH)‐type granulocytes (Figure 1) and 0.007% PNH‐type erythrocytes, with a plasma TPO level of 765 pg/mL. Based on these findings, the patient was diagnosed with immune AA.

**FIGURE 1:**
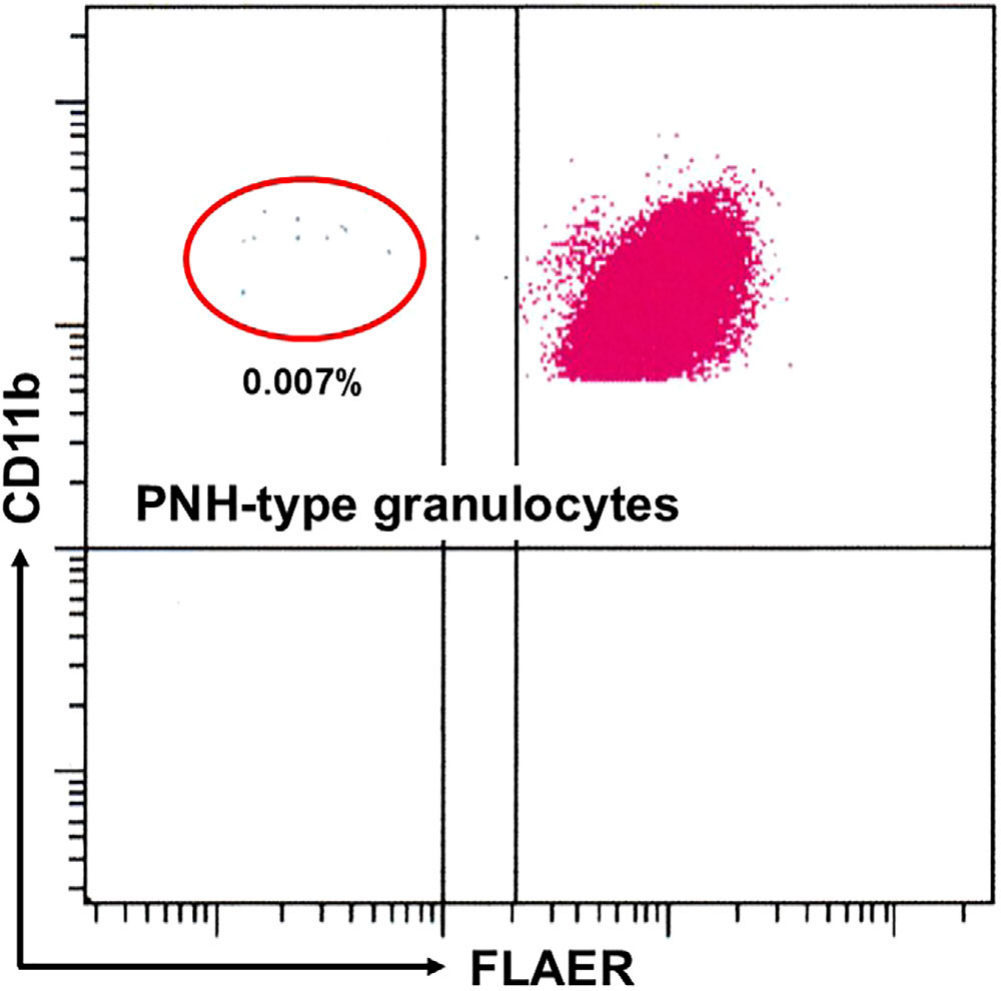
PNH‐type granulocytes detected in this case. PNH, paroxysmal nocturnal hemoglobinuria.

Her gastric cancer was difficult to resect endoscopically, thus necessitating surgical resection. However, her platelet count was below 20,000/µL, making surgical resection difficult due to thrombocytopenia. Therefore, the treatment of AA was deemed necessary. The AA severity was classified as severe according to the Camitta criteria [4], prompting consideration of IST, including ATG. However, due to concerns about the progression of untreated gastric cancer, ATG was avoided, and AA treatment was started with CsA and ROMI (10 µg/kg/week). The CsA blood concentration was adjusted to the target C2 level of 600 ng/mL. Following the initiation of CsA and ROMI, the platelet count and reticulocyte count increased rapidly, thus eliminating the need for red blood cell transfusions (Figure 2). A partial hematological response was achieved one month after treatment initiation. No deterioration in the renal function was observed after the initiation of CsA. No gastric cancer metastases were detected during CsA or ROMI therapy. Laparoscopic pyloric gastrectomy was performed 63 days after IST initiation. No bleeding or thrombotic complications were noted. Following surgery, the ROMI dosage was reduced, and treatment with CsA and ROMI was continued.

**FIGURE 2:**
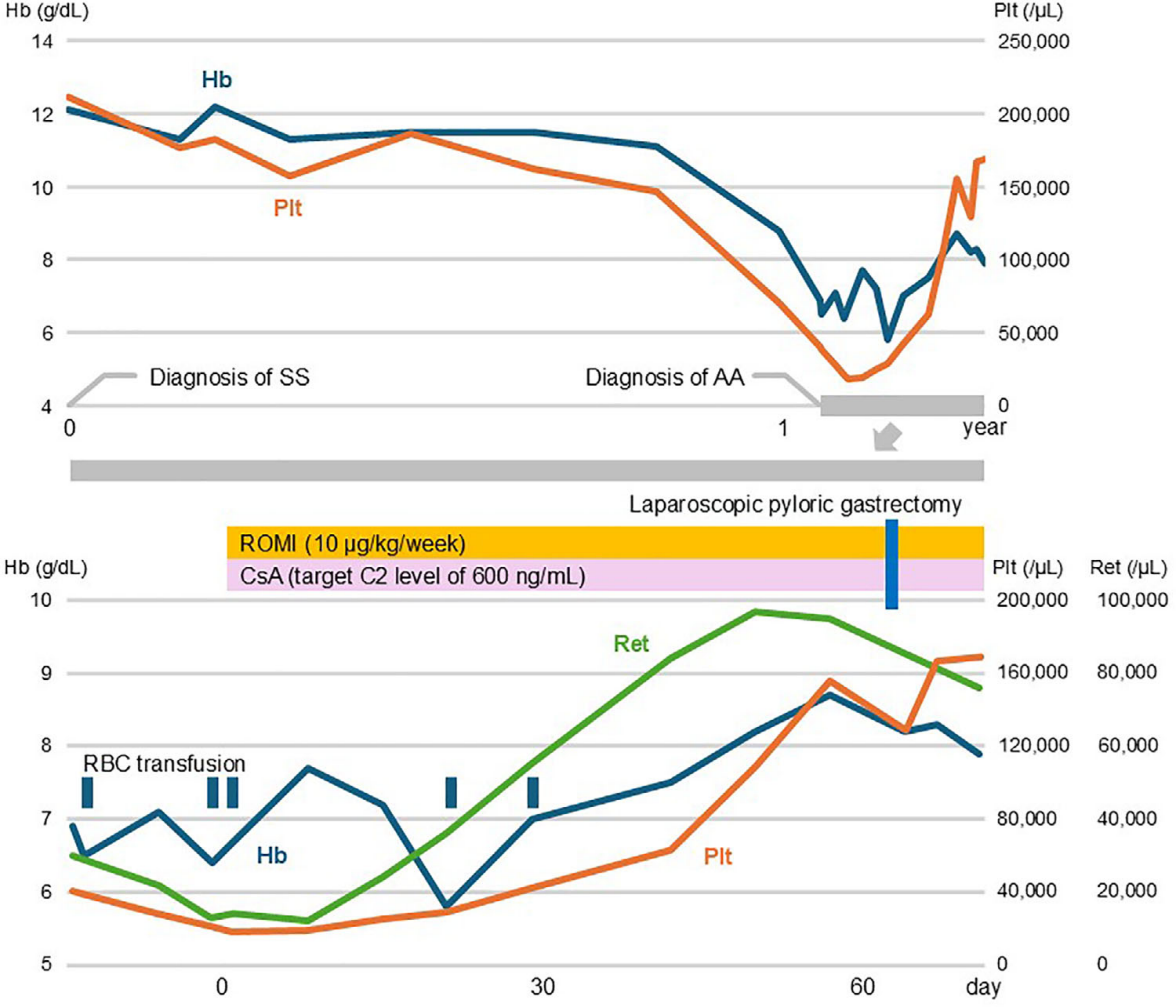
Clinical course after the diagnosis of SS. After the diagnosis of SS, anemia progressed, and endoscopy revealed gastric cancer. Thrombocytopenia progressed, resulting in pancytopenia. After the diagnosis of AA, CsA and ROMI were initiated. Following the initiation of CsA and ROMI, the platelet and reticulocyte counts increased rapidly, thus eliminating the need for red blood cell transfusions. Laparoscopic pyloric gastrectomy was performed 63 days after initiating CsA and ROMI. AA, aplastic anemia; CsA, cyclosporine A; Hb, hemoglobin; Plt, platelet; RBC, red blood cell; Ret, reticulocyte; ROMI, romiplostim; SS, Sjögren's syndrome.

## 3 Discussion

We herein report a case of immune‐mediated AA complicated by gastric cancer that was successfully treated with CsA and ROMI as a bridging therapy before curative surgery. This patient had untreated gastric cancer at the time of AA diagnosis, and surgery was difficult because of thrombocytopenia. However, CsA and ROMI were effective, resulting in a rapid increase in the platelet count, thereby making surgery for gastric cancer possible. PNH‐type cells can be useful markers for determining the treatment strategies for AA with malignant tumors. CsA and ROMI may be effective treatment options for the rapid improvement of blood counts in patients with AA complicated by malignant tumors.

Because the AA in this case was classified as severe, IST, including ATG, was considered. However, ATG was avoided due to concerns about the progression of untreated gastric cancer. Previous studies have reported that ATG‐based IST in AA increases the long‐term risk of several malignancies [5, 6, 7]. However, research on IST for AA with untreated malignant tumors is limited. In most prospective studies, concomitant malignancies were excluded from the clinical trials. We previously reported a study of AA secondary to chemoradiotherapy for cancer [8]. In this study, two of four patients who received IST and were in non‐complete remission of their original cancers showed progression of their cancers. There is some potential concern that IST increases the risk of cancer aggravation by suppressing the immune surveillance of cancers. In particular, the use of ATG, which has a pan T‐cell suppressive effect, needs to be determined carefully while considering the risk‐benefit balance of IST.

A phase II/III trial using CsA and ROMI in IST‐naïve patients with AA demonstrated an overall response rate of 23.1% at Week 14, and 46.2% at Week 27 in patients with severe AA [9]. Several studies have also reported the efficacy of ROMI in AA patients refractory to EPAG [10, 11, 12], and ROMI may have a potent hematopoietic effect, although direct comparisons with EPAG are lacking. The presence of a minor PNH‐type clone supported the immune‐mediated nature of AA and the rationale for IST [13, 14]. In addition, IST has been reported to be effective even in patients with PNH‐type cells at levels of 0.003%–0.01% [15]. Based on these findings, CsA and ROMI were selected to prioritize platelet recovery and thus make it possible to perform timely surgical resection. CsA and ROMI may be effective treatment options for a rapid improvement of the blood counts in patients with AA complicated by malignant tumors. Furthermore, PNH‐type cells can serve as predictors of the response to IST and they are useful markers for determining the treatment strategies for AA in malignant tumors. As this is a single case, the generalizability of this approach remains limited.

In conclusion, the administration of CsA and ROMI as a bridging therapy to curative surgery is considered to be a feasible treatment option in patients with AA complicated by malignant tumors. In this case, CsA and ROMI rapidly improved the blood counts, thus making surgery possible for gastric cancer. Therefore, the close monitoring of gastric cancer recurrence is necessary. Further studies are needed to clarify the optimal treatment strategy for AA with malignant tumors.

## Author Contributions

Noriharu Nakagawa designed the study and prepared the manuscript. Masahide Yamazaki and Kohei Hosokawa reviewed the manuscript. Noriharu Nakagawa treated the patient. All the authors critically reviewed and checked the final version of the manuscript.

## Funding

The authors have nothing to report.

## Ethics Statement

Written informed consent was obtained from the patient. This study was approved by the Ethics Committee of Keiju Medical Center (no. 2025‐5‐1).

## Conflicts of Interest

Kohei Hosokawa received honoraria from Kyowa Kirin Co. Ltd.

## Data Availability

No datasets were generated or analyzed during this case report.
